# Extract from the Records of the Atlanta Medical Society

**Published:** 1856-03

**Authors:** Eben Hillyer


*Extract from the records of the Atlanta Medical Society.* By EBEN HILLYER, M. D., Secretary. Jan. 10, 1856.

*Chloroform in a Case of Eclampsia.*—Dr. Darnall reported the following case:—He was called at 7 o'clock, Nov. 28th, 1855, to see Mrs. W——, etat 18 years, first child; found her suffering from intense pain in the right side of the head and right eye; with a frequent shivering, without a sense of coldness; full, strong pulse; not much increased in frequency; with dimness of sight; the face, arms and legs were considerably swollen; there was, also, evident contraction of the uterus. Upon examination per vagina, he found the os uteri quite thin, and very slightly dilated; evidently showing she had reached the full term of her first pregnancy. Her general health had been very bad from an early period after conception, until now. In short, she had suffered all the ills "woman is heir to" during gestation; consequently, she was pale and anemic. The symptoms above enumerated, impressed him with the belief that convulsions would soon come on unless prevented by an active course of treatment. Accordingly, he bled her—about thirty ounces, (by estimate.) The pain in the head and eye was not relieved to the extent he expected by the bleeding, notwithstanding its apparent copiousness.

A short time previous to the Doctor's arrival, she had taken a full dose of Chapman's peristaltic persuaders. He, therefore, did not give any more cathartic medicine—deeming it best to wait a reasonable time for the operation of the dose she had already taken. Feeling a good deal of solicitude for his patient, he remained with her until 10½ o'clock. She was then sleeping, and as far as he could judge, she was easy. But in less than an hour after he left, she was attacked with a severe convulsion. Dr. Darnall was immediately sent for, as was, also, Professors J. G. and W. F. Westmoreland. He found Dr. J. G. Westmoreland with her, who had bled her again (by estimate) ℥xvi to ℥xviij. She was then in a convulsion, and he opened the orifice, and allowed xviii℥ or ℥xx more to flow out. The convulsions ceased for four and a half hours, and then returned. Again they opened the orifice, and allowed ℥xx to flow; and again the convulsions ceased for three hours, and returned. The question now was, what could they do to save their patient. They had carefully watched the progress of the labor; and the condition of the cervix uteri, which was not in a condition that would allow them to turn and deliver. The use of the forceps was of course precluded for the same reason. Dr. W. F. Westmoreland proposed to give her chloroform, which was agreed to; accordingly, they gave her xxx gtts. every half hour in a table spoon full of water, until the convulsions ceased. The same dose was then continued for one hour or more. She continued to rest well ten hours, when efficient labor came on, and she was delivered of a dead fœtus, weighing nine pounds, without manual assistance, about forty hours after she was attacked. Conciousness did not return until Sunday, some forty-eight hours after delivery. She is now convalescent.

Dr. D. remarked that this was the first case of the kind in which he had seen chloroform used, and judging from its effects, he should use it with much confidence in all similar cases; that is, after the patient had been sufficiently depleted by the lancet. He was disposed to believe, under such circumstances, that it was equal, if not superior, to opium and its various preparations, in combination with tart. ant. et potass.

Dr. Logan remarked, that recently he had a case of retention of urine, which was promptly relieved by the external application of chloroform over the region of the bladder and loins.

Dr. Hillyer reported a case of flatulent colic, which was relieved by chloroform. He was called to see Mr. J. P. S. at 8 o'clock, Dec. 10th, 1855; found his patient violently attacked with colic; he had been suffering near an hour when he arrived; he was unable to speak; pulse very irregular, about sixty to the minute—two beats and then an intermission, then two or three beats in quick succession, then another intermission. When first attacked, he complained of great pain in the stomach; his breathing was irregular, and somewhat laborious. When asked where the pain was located, he placed his hand upon the epigastric region. The friends of the patient informed Dr. H. that he was subject to similar attacks, but none were ever as violent as this. Dr. H. thought him in great danger, and unless immediate relief could be obtained, he would die. He gave him thirty drops of chloroform in a teaspoon full of water, and applied a large sinapism over the stomach and abdomen; in ten minutes he was greatly relieved; and in half hour, his pulse had become natural, and the patient said he was in no pain whatever.

[Since the above case was reported to the Society, I was called to the same patient, in a similar attack. The chloroform was administered as before, with as prompt relief. This agent has not been used internally, as an antispasmodic, to a very great extent. From my observation, I think it superior to any agent we have. I have known as much as a drachm taken at one dose, without any evil result.—*Secretary.*]

January 18th.

*Case of Arm Presentation.*—Dr. John G. Westmoreland was called to see Jane, colored girl, at 8 o'clock, on the 5th April, in labor; upon examination, could not make out the presentation. The cervix high in the pelvis, and dilated to the size of half dollar; pains seldom, and but of little force. At 9 the pains had increased in force and frequency, still not severe. The os uteri, statu quo, through which was protruded the membranes containing quite a quantity of liquor amnii; still could not make out the presentation. He determined to reach the fœtus if possible; and in order to do so, was compelled to introduce the whole hand into the vagina. He could place his finger in contact with some part of the child, but from its elevated position, and the large quantity of water, (which he did not then wish to discharge,) he could not determine positively the part presenting. It was not long before the pains increased; the waters advanced; the os became more dilated, and from his manipulation, the membranes ruptured, he could then distinguish the hand and fore arm, and what he supposed to be the thigh; the hand was the most prominent presenting part, and soon made its appearance in the vagina. He returned it, and kept it above the brim of the pelvis during several pains, hoping that the hip and buttocks would descend, and engage in the pelvis, but in this he was disappointed. Dr. W. then called his brother and Doctors Boring and Parker in consultation. It was determined to obtain and bring down the knee, which was done by Dr. W. F. Westmoreland, with the aid of the blunt hook. The delivery was tedious, and was not complete until about 7 o'clock, P. M. The child was dead; the funis presented with the head. The woman finally did well. The left side of the child presented to the left side of the mother. He states that in his examination, he detected what he supposed to be the placenta, low down in the left side of the uterus; and his conclusions are, that there would have been a natural breech presentation, had not the attachment of the placenta at that part of the womb deviated, the presenting foot from its proper direction.

